# Proteomic responses to hypoxia at different temperatures in the great scallop (*Pecten maximus*)

**DOI:** 10.7717/peerj.871

**Published:** 2015-03-31

**Authors:** Sébastien Artigaud, Camille Lacroix, Joëlle Richard, Jonathan Flye-Sainte-Marie, Luca Bargelloni, Vianney Pichereau

**Affiliations:** 1 Laboratoire des Sciences de l’Environnement Marin, LEMAR UMR 6539 CNRS/UBO/IRD/Ifremer, Université de Bretagne Occidentale, Institut Universitaire Européen de la Mer, Plouzané, France; 2 Department of Comparative Biomedicine and Food Science—Agripolis—Viale dell’Università 16, Legnaro, Padova, Italy

**Keywords:** Proteomic, Hypoxia, Temperature, Bivalves, Non-model, Marine biology

## Abstract

Hypoxia and hyperthermia are two connected consequences of the ongoing global change and constitute major threats for coastal marine organisms. In the present study, we used a proteomic approach to characterize the changes induced by hypoxia in the great scallop, *Pecten maximus*, subjected to three different temperatures (10 °C, 18 °C and 25 °C). We did not observe any significant change induced by hypoxia in animals acclimated at 10 °C. At 18 °C and 25 °C, 16 and 11 protein spots were differentially accumulated between normoxia and hypoxia, respectively. Moreover, biochemical data (octopine dehydrogenase activity and arginine assays) suggest that animals grown at 25 °C switched their metabolism towards anaerobic metabolism when exposed to both normoxia and hypoxia, suggesting that this temperature is out of the scallops’ optimal thermal window. The 11 proteins identified with high confidence by mass spectrometry are involved in protein modifications and signaling (e.g., CK2, TBK1), energy metabolism (e.g., ENO3) or cytoskeleton (GSN), giving insights into the thermal-dependent response of scallops to hypoxia.

## Introduction

Temperature and oxygen availability are two of the most prominent abiotic factors impacting marine organisms in natural environments. Due to global change, both are changing dramatically (Harley et al., 2006; Diaz & Rosenberg, 2008). In this context, understanding how hypoxia and temperature affect physiology of marine organisms is crucial. Temperature variations affects the physiology of marine organisms, modifying their responses to other stressors (Pörtner, 2005). Hypoxia also directly impact organisms ability to respond to temperature variations, as thermal windows are limited by the capacity of marine organisms to sustain a rise in aerobic scope (Pörtner, 2001). A decrease in molecular oxygen (O_2_) is of major concern for organisms since O_2_ is required by most animals in order to achieve essential metabolic processes. However, all organisms do not display the same responses to an oxygen decline in their environment. “Oxy-regulators” are animals for whom oxygen uptake rate remains unaffected by the changes in environmental oxygen concentration, whereas oxygen uptake rate of “oxy-conformers” shows a decrease with decreasing environmental oxygen concentration (Grieshaber et al., 1994). Responses to hypoxia can be further characterized by assessing the value of the oxygen critical point (PcO_2_), a point where an oxy-regulator becomes an oxy-conformer (Grieshaber, Kreutzer & Pörtner, 1988). From a metabolic perspective, this threshold reflects a change from an aerobic- towards an anaerobic-pathway of energy production (Pörtner & Grieshaber, 1993). In the classic anaerobic pathway, pyruvate is catalysed by the lactate dehydrogenase into lactate, reoxydating NADH + H^+^ into NAD^+^ (Grieshaber et al., 1994). Among the invertebrates five other pyruvate reductases have been found, usually called opine dehydrogenases. They catalyse the reductive condensation of pyruvate with an amino acid resulting in the synthesis of an imino acid (Grieshaber et al., 1994). In the marine mollusc *Pecten maximus*, octopine dehydrogenase is present and more active than the lactate dehydrogenase, allowing the synthesis of octopine from the condensation of pyruvate and arginine (Zammit & Newsholme, 1976; Grieshaber et al., 1994).

Marine mollusk bivalves such as the great scallop *Pecten maximus* are sessile animals and are often exposed to changes in temperature and oxygen concentration in their natural environment. They play key roles in coastal ecosystems, as major calcium and carbon accumulators and as links between primary producers and organisms at higher trophic levels in marine food webs. *P. maximus*is a bivalve mollusk, distributed along the North Atlantic coast from the North of Norway to the South of Portugal and off the west coast of Africa (Brand, 2006). *P. maximus* is an economically important species, representing almost 80% of European wild harvested scallops (Brand, 2006). Aquaculture of *P. maximus* is expanding, especially in France and Ireland where hatchery-produced seed is used to enhance the production in the wild (Brand, 2006). In our area of study (Bay of Brest, France) *P. maximus* usually experience temperatures from around 10 °C in winter to 18 °C in summer (data from Somlit-Brest, Coastal time-series station; 10 m depth) and can be exposed to severe local hypoxia during summer following eutrophication events. In a recent study from our group, *P. maximus* was found to be a strong oxy-regulator but its ability to regulate decreases when temperature rises (Artigaud et al., 2014c).

In order to further investigate the response of *P. maximus* to hypoxia at different temperatures, 2DE-base proteomics were used. Proteomics and molecular biology approaches have started to spread in the field of marine biology, and some proteomic studies have been conducted in bivalves, either evaluating effects of different stressors or comparing populations in the field (recently reviewed in Sheehan & McDonagh, 2008; for example: Tomanek, 2011; Campos et al., 2012; Artigaud et al., 2014b). Proteomics is a method of choice as changes in protein abundances represent modifications of the molecular phenotype of the cells, and therefore functional changes (Feder & Walser, 2005). Despite its physiological relevance, 2D-based proteomics is however intrinsically biased towards highly abundant soluble proteins. Other studies have investigated the impacts of hypoxia in bivalves using other approaches such as transcriptomic or targeted immuno-assay approach (for example, David et al., 2005; Le Moullac et al., 2007; Sussarellu et al., 2010; Guévélou et al., 2013). To our knowledge, the work presented here is the first study exploring proteomic responses in bivalves to hypoxia at different temperatures.

The present study focuses on the responses of *P. maximus* to a short (24 h) but severe hypoxic stress at 3 different temperatures, reflecting their natural ambient range (10 °C and 18 °C) in the Bay of Brest and a more stressful (25 °C) condition. To assess the differences of regulation at three different temperatures, protein expression between normoxic and hypoxic conditions were compared using two-dimensional electrophoresis (2-DE). Significantly deregulated proteins were analyzed by tandem mass spectrometry. Furthermore, as hypoxia is closely linked to the energetic metabolism, we also used the arginine content and the octopine dehydrogenase (ODH) activity as proxies of the anaerobic metabolism activation.

## Materials and methods

### Animal experimentation

In March 2013, 100 six-months-old scallops (average length ± standard deviation: 35.5 ± 2.6 mm) were provided by the Tinduff hatchery (Bay of Brest, France). Animals were split into 3 homogeneous groups (approximately 30) and acclimated in separate flow-through tanks containing sand-filtered, air-saturated seawater maintained at 10 °C (ambient field temperature). After one week of acclimation, two of the tanks were heated (1 °C per day) until they reached 18 °C or 25 °C, respectively. Once at the desired temperature, animals were maintained at least one week before performing hypoxia challenges. Temperatures in the three tanks were monitored continuously during the whole experiment using an autonomous temperature logger (EBRO, Ingolstadt, Bayern, Germany). Temperatures were maintained in each tank using pumps and O_2_-saturation was kept at 100% until hypoxia challenges. Seawater parameters (temperature, pH, salinity and O_2_ levels; available as File S1) and mortality were assessed daily in each tank during the whole experiment and a day-night (12/12) light cycle was maintained.

### Hypoxia challenge and sampling

For the hypoxia challenge, scallops were placed in another thermo-regulated tank in which O_2_ concentration was controlled by a computer through nitrogen injection. A feedback was provided to the computer by an O_2_ sensing probe (FDO 925-3; WTW, Oberhayern, Germany) placed in the tank and nitrogen flow was adjusted in order to maintain O_2_ level to a set-value. Once in the O_2_ controlled experimental tank, animals were acclimated for at least 2 h before oxygen level was decreased (within 1 h) to the given set-value. To ensure that animals were in hypoxic environment, they were exposed to a level of oxygen below their PcO_2_ at the given temperature. PcO_2_ is the oxygen critical point, a point where an oxy-regulator becomes an oxy-conformer. PcO_2_ in these conditions were determined in a previous study (Artigaud et al., 2014c). Depending on their temperature of acclimation, animals were exposed as follows: animals acclimated at 10 °C were exposed to 7.6% ± 0.3 of O_2_ saturation (PcO_2_ at 10 °C: 18.3%), animals at 18 °C were exposed at 7.7% ± 0.2 (PcO_2_ at 18 °C: 23.8%), and animals at 25 °C were exposed at 14% ± 0.3 (PcO_2_ at 25 °C: 36.1%). Animals were left for 24 h in hypoxic seawater before being sampled.

After 24 h of hypoxia challenge, animals were quickly dissected, adductor muscles and gills were snap-frozen in liquid nitrogen and kept at −80 °C until further analysis. Care was taken to minimize air exposure during the sampling. Scallops acclimated at the same temperatures but maintained in normoxic conditions were dissected in similar conditions at the same time.

### Determination of ODH activity and arginine content

Adductor muscles were used for measurements of anaerobic metabolism since glycogen reserves, the main source of energy for anaerobic metabolism, are mainly found in this tissue. Frozen muscles were crushed with a mixer mill (MM400; RETSCH, Haan, Germany) and kept frozen using liquid nitrogen. For octopine dehydrogenase (ODH; EC1.5.1.11) activity, muscle powder was homogenized on ice with an Ultra-Turrax (Model Pro 200; PRO Scientific Inc., Oxford, Connecticut, USA) after adding 6 volumes (w/v) of homogenizing-buffer (20 mM Tris–HCL, 1 mM EDTA, 1 mM DTT, pH 7.5). The homogenate was centrifuged for 15 min at 12 000 g and 4 °C. ODH activity was determined in supernatants according to Livingstone et al. (1990). The decrease in absorbance of NADH_2_ at 340 nm was recorded over 10 min in 15 s intervals in a fluo-spectrometer microplate reader (POLARStar Omega, BMG Labtech, Offenburg, Germany) at 25 °C. No activities were recorded in the absence of L-arginine, thus showing the lack of lactate dehydrogenase activity.

For determination of arginine contents, muscle powder was homogenized on ice with an Ultra-Turrax after adding 6 volumes (w/v) of 0.5 M perchloric acid. The homogenate was centrifuged for 15 min at 12 000 g and 4 °C and the supernatant of each sample was neutralized with 2 M KOH and centrifuged for 5 min at 12 000 g and 4 °C. Arginine contents were determined enzymatically according to Gaede & Grieshaber (1975), except that the incubation time in the presence of a purified ODH (purchased from Sigma-aldrich) was prolonged to 2 h, to allow complete reaction of all arginine in the samples. The fluorescence of NADH_2_ was measured in a fluo-spectrometer microplate reader (355 nm excitation/460 nm emission) and arginine contents were deduced from standards of arginine (0–120 µM).

Statistical analyses of arginine content and ODH activity were conducted in R (R Core Team, 2013). Normality was checked with Shapiro’s test and homoscedasticity using Bartlett’s test. When these criteria were met, Student’s *t*-test and ANOVA were used to compare differences between samples, and Tukey’s test was used as a post hoc. When normality could not be established, a permutation ANOVA was used according to Anderson & Legendre (1999).

### Proteins extraction

Gills were chosen as the experimental tissue for comparing proteins abundance. Gills are directly in contact with water and responsible for oxygen extraction, gene chip studies have shown that bivalve tissues directly in contact with the external environment are more responsive to environmental perturbation than internal tissues (Clark et al., 2013). Hence, proteomics profiles of the gills can act as an effective proxy of the early whole-animal response.

Frozen gills were crushed as described above for the determination of ODH activity and arginine content. For each animal, 100 mg of the obtained powder was homogenized in 100 mM Tris–HCl (pH 6.8) with 1% of Protease inhibitor mix (GE Healthcare, Little Chalfont, UK), centrifuged (50 000 g, 5 min, 4 °C) and supernatants were transferred to new tubes. Nucleic acids were then removed (nuclease mix, GE Healthcare, following manufacturer’s instructions). Samples were precipitated at 4 °C using TCA 20% (1/1:v/v, overnight). After centrifugation (20 000 g, 30 min, 4 °C), pellets were washed with 70% acetone and re-suspended in Destreak buffer (GE Healthcare, Little Chalfont, UK) containing 1% ampholytes (IPG Buffer, pH 4–7; GE Healthcare, Little Chalfont, UK). Protein concentrations were determined using a modified Bradford assay (Ramagli, 1999), and all samples were adjusted to 200 µg of proteins in 250 µl.

### Two-dimensional electrophoresis

Prior to isoelectric focusing, IPG strips (pH 4–7, 13 cm; GE Healthcare, Little Chalfont, UK) were passively rehydrated with 250 µl of protein solution in wells for 14 h. Isoelectric focusing was conducted using the following protocol: 250 V for 15 min, 500 V for 2 h, gradient voltage increased to 1 000 V for 1 h, gradient voltage increased to 8 000 V for 2,5 h, 8 000 V for 3 h, and finally reduced to 500 V (Ettan IPGphor3; GE Healthcare, Little Chalfont, UK). To prepare for the second-dimension SDS-PAGE, strips were incubated in equilibration buffer (50 mM Tris–HCl pH 8.8, 6 M urea, 30% glycerol, 2% SDS and 0.002% Bromophenol Blue) for two 15 min periods, first with 1 g l^−1^ dithiothreitol and then with 48 g l^−1^ iodoacetamide. IPG strips were placed on top of 12% polyacrylamide gels, which were run in thermo-regulated electrophorese unit at 10 °C (SE 600 Ruby; Amersham Biosciences, Amersham, UK) at 10 mA per gel for 1 h and then 30 mA per gel until complete migration. Gels were subsequently stained with “Blue Silver” (Candiano et al., 2004) and destained with Milli-Q water for 48 h. The resulting gels were scanned with a transparency scanner (Epson Perfection V700; Epson, Suwa, Nagano, Japan ) in gray scale with 16-bit depth and a resolution of 400 dpi.

### Gel image analysis and statistical analysis of proteins’ abundance

Images were aligned and spots were detected and quantified using the Progenesis SameSpots software (version 3.3, Nonlinear Dynamics) using the automated algorithm. All detected spots were manually checked and artifact spots were removed. Data were exported as raw values and statistical analyses were conducted in R (R Core Team, 2013) using the prot2D (Artigaud, Gauthier & Pichereau, 2013) and limma packages (Smyth, 2004) from the Bioconductor suite (Gentleman et al., 2004). Data were normalized (quantile normalization) and the samples were paired compared between hypoxia and normoxia conditions using moderated *t*-test at each temperature with 5 replicates per condition. For comparisons, we used a moderated *t*-test, which is a modified t-test, for which the standard errors have been moderated across spots, increasing the reliability of the test (Smyth, 2004; Artigaud, Gauthier & Pichereau, 2013). Once the values of moderated *t*-test were calculated, a global correction by false discovery rate (fdr) was applied, in order to take into account multiple comparisons issues and paired-comparison correction. Spots with an fdr threshold lower than 0.1 and an absolute fold change higher than 2 were considered as differentially expressed.

### Mass spectrometry

Proteins that changed significantly in abundance in response to hypoxia for the different temperatures were excised from gels and prepared for analysis by mass spectrometry (MS). Gel pieces were first washed in 50 mM ammonium bicarbonate (BICAM), and then dehydrated in 100% acetonitrile (ACN). Gel pieces were vacuum dried, and rehydrated with BICAM containing 0.5 µg of Porcine recombinant trypsin (sequencing grade; Promega, Madison, Wisconsin, USA), and incubated overnight at 37 °C. Peptides were extracted from the gels by alternative washing with 50 mM BICAM and ACN, and with 5% formic acid and ACN. Between each step, the supernatants were pooled, and finally concentrated by evaporation using a centrifugal evaporator (Concentrator 5301; Eppendorf, Hamburg, Germany). Samples were resuspended in Trifluoroacetic acid (TFA; 0.1% in water). Peptide solutions were mixed with the *α*-Cyano-4-hydroxycinnamic acid (HCCA, 10 mg/ml of a ACN/TFA/water (60/4/36:v/v/v) solution), and spotted on a polished steel target using the dried droplet method. Peptides were analyzed by Matrix-Assisted Laser Desorption Ionization Time-Of-Flight tandem mass spectrometry (MALDI TOF-TOF) in positive ion reflector mode, using an Autoflex III (Bruker Daltonics, Billerca, Massachusetts, USA) mass spectrometer. The flexControl software (v3.0; Bruker Daltonics, Billerca, Massachusetts, USA) was programmed to acquire successively PMF spectra and MS/MS from the dominant peaks. Mass spectra were analyzed with flexAnalysis (v3.0; Bruker Daltonics, Billerca, Massachusetts, USA) by applying the following conditions: TopHat algorithm for baseline subtraction, Savitzky-Golay analysis for smoothing (0.2 *m*/*z*; number of cycles: 1) and SNAP algorithm for peak detection (signal-to-noise ratio: 6 for MS and 1.5 for MS/MS). The charge state of the peptides was assumed to be +1. Porcine trypsin fragments were used for internal mass calibration.

Proteins were identified with the PEAKS software (v 5.3; Bioinformatics Solutions, Waterloo, Ontario, Canada), using MS/MS-based identification and *de novo* peptide sequencing. A custom-made expression sequence tags (EST) database (see below) was used with the following search parameters: carbamidomethylation of cysteine was set as a fixed modification; oxidation of methionine and phosphorylation of serine, threonine or tyrosine were set as variable modifications; one missing cleavage during trypsin digestion was allowed. Protein identification was considered as unambiguous when a minimum of two peptides matched with a minimum score of 20. False discovery rates were also estimated using a reverse database as decoy.

The EST database (available as http://figshare.com/articles/Pecten_maximus_EST_database/1328466) was constructed by combining *P. maximus* sequences from Illumina RNAseq sequenced from mantle tissues (Artigaud et al., 2014a), and from hemocyte cells (Pauletto et al., 2014). Overall, the database included a total of 252 888 *P. maximus* EST. EST sequences were annotated by homology searches against a non-redundant protein database using the Blast algorithm from NCBI with an e-value cut-off of 1⋅e^−10^ (Altschul et al., 1997).

## Results and Discussion

### General patterns of enzymatic and proteomic response

In natural environments, marine mollusks are subjected to an array of stresses among which elevated temperature and hypoxia are one of the most significant and the most historically studied (for example: Krogh, 1916; Taylor & Brand, 1975; De Zwaan & Wijsman, 1976; Bayne & Livingstone, 1977; Taylor, Butler & Al-Wassia, 1977). The aim of this study was to estimate the anaerobic metabolism through enzymatic assays and to decipher the proteomic signatures of hypoxia in animals exposed to three different temperatures: 10, 18 and 25 °C. No mortality was observed during the exposure at 10 or 18 °C, but at 25 °C under hypoxic conditions 50% mortality occurred, reflecting a severe stress.

The transition to hypoxia should force animals to shift their metabolism towards anaerobic metabolism. The main fermentative metabolism in mollusks is the octopine dehydrogenase (ODH) pathway, that catalyses the condensation of glycolytic pyruvate and arginine into octopine, the final fermentative product (Storey & Storey, 2004). This reaction allows the restoration of the pyridines reduced during glycolysis (i.e., NADH_2_). As an attempt to evaluate the metabolic state of animals in our experimental conditions, the arginine contents and ODH activities were assayed in all conditions.

**Figure 1:**
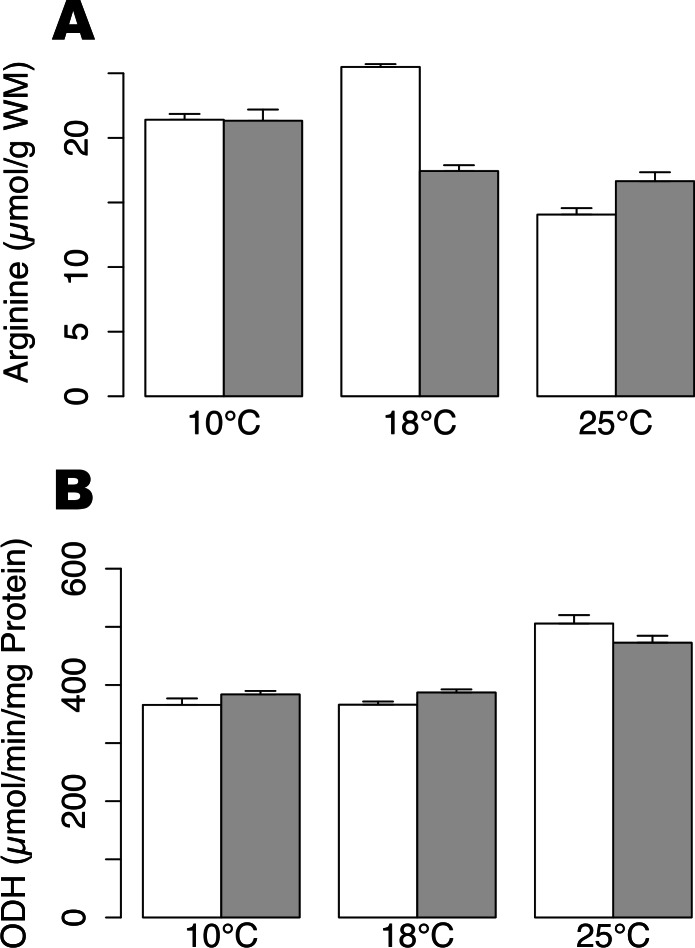
Arginine contents and ODH activity in *Pecten maximus* muscles. Arginine contents (A) and octopine dehydrogenase activity (B) at three different temperatures (10 °C, 18 °C, 25 °C) in normoxic condition (blank) and following 24 h hypoxia (grey) in muscle tissue of *Pecten maximus*. Vertical bars represent standard error of the mean.

Arginine contents were not significantly different between hypoxic and normoxic conditions at 10 °C and 25 °C. Arginine content decreased significantly during hypoxia at 18 °C (Fig. 1A; *t*-test, *p*-value < 0.01) and could indicate a fermentative shift, as arginine is used in order to product octopine, the final product of anaerobic metabolism. In animals maintained under normoxic conditions, arginine concentrations were significantly lower at 25 °C compared to 10 °C and 18 °C (Tukey HSD, *p*-value < 0.01). The ODH activity showed a significant increase at 25 °C, as compared to 10 and 18 °C (Fig. 1B; permutation ANOVA, *p*-value = 0.001), but no significant difference was shown between animals subjected or not to hypoxia at any temperature (*t*-test, *p*-value > 0.05).

**Figure 2:**
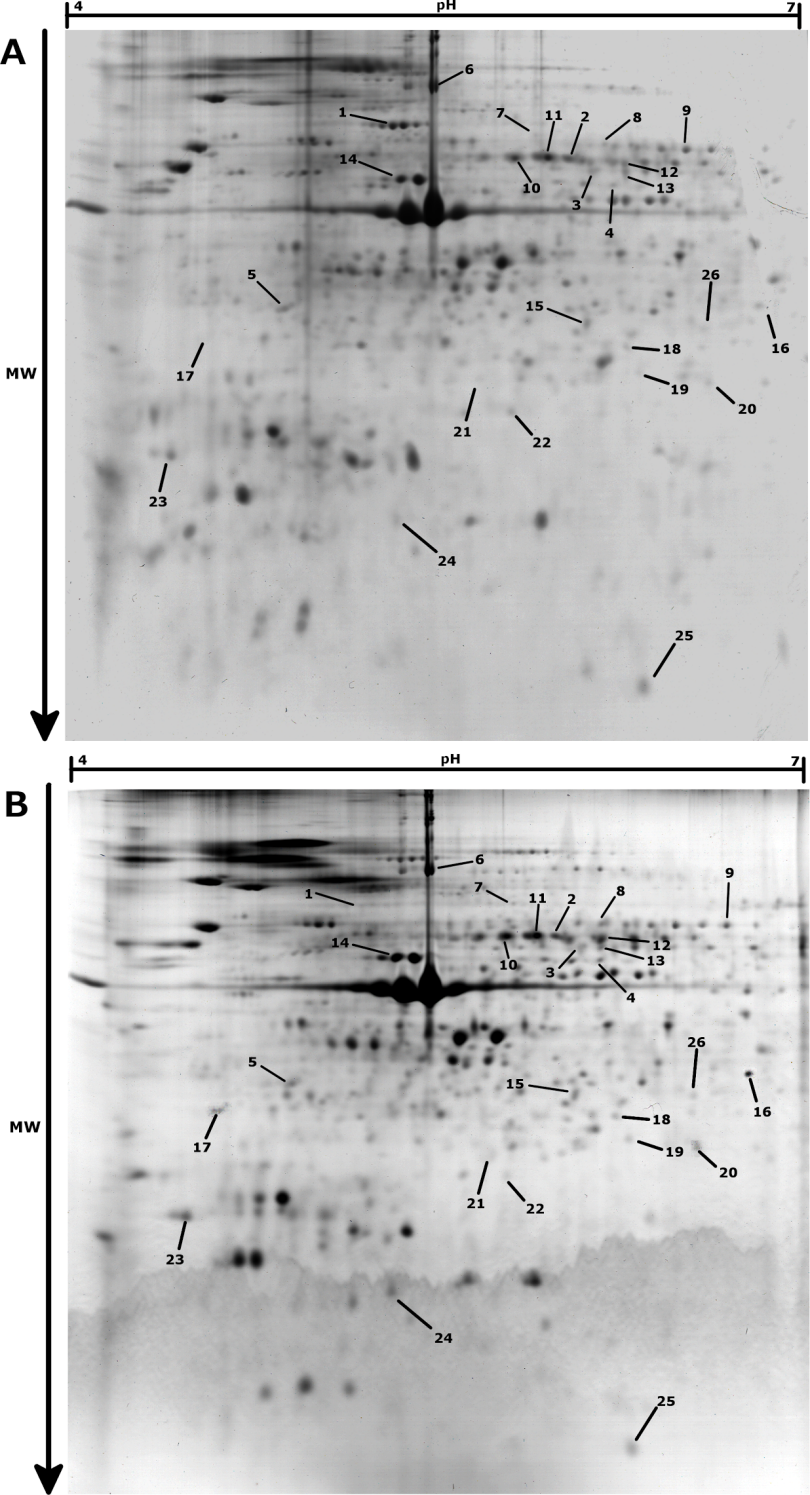
Representative 2-DE gels (pH 4–7, SDS-PAGE 12%) for *Pecten maximus* gills proteins in Normoxic (A) and Hypoxic (B) conditions at 18 °C. Spots showing significant differential accumulation are arrowed.

In the proteomic analysis, 647 spots were observed across the 30 gels analyzed (Fig. 2). Changes in protein contents were examined between normoxia and hypoxia conditions, for the three different temperature treatments (10 °C, 18 °C and 25 °C). At 10 °C, no significant change in protein abundances between normoxia and hypoxia conditions were observed (paired moderate *t*-test, fdr < 0.1, absolute fold change >2), suggesting no major adjustment to hypoxia in the gills of animals acclimated at 10 °C. At 18 °C, 16 protein spots were found to be significantly differentially accumulated between hypoxic and normoxic conditions, 10 of which being up-regulated during hypoxia whereas 6 were down-regulated (Table 1). At 25 °C, only 1 protein displayed a significantly greater abundance and 10 proteins were at lower abundance during hypoxia. Surprisingly, only one identified protein (spot 7; Fig. 2, Table 1), displayed a similar accumulation profile under hypoxic conditions at both 18 °C and 25 °C.

**Table 1:** Log_2_ Fold Change (FC) for the normalized volumes of spots differentially expressed. List of spots issued from two-dimensional electrophoresis of gills proteins from Pecten maximus maintained at 3 different temperatures (10 °C, 18 °C and 25 °C) in hypoxic and normoxic conditions. Values correspond to the Log_2_ Fold Change (FC) for the normalized volumes of spots between animals in Hypoxic and Normoxic conditions. Bold indicate the values identified as differentially accumulated (paired moderate *t*-test, fdr < 0.1, absolute Log_2_ FC > 1).

	Log_2_ FC (Hypoxia/Normoxia)		
Spot	10 °C	18 °C	25 °C
1	−0,13	**−1,24**	−0,84
2	−0,73	**−1,74**	−0,7
3	−0,8	**−1,92**	−0,49
4	−0,26	**−1,23**	−0,07
5	−0,48	**−1,16**	0,53
6	−0,31	−0,16	−1,02
7	0,22	**−1,19**	**−1,15**
8	0,03	−0,28	**−1,16**
9	−0,3	−0,74	**−1,89**
10	0,14	−0,31	**−1,32**
11	−0,01	−0,83	**−1,98**
12	−0,08	−0,39	**−1,07**
13	−0,21	−0,62	**−1,1**
14	−0,07	−0,15	**−1,02**
15	−0,79	0,35	**−1,72**
16	0,12	**1,31**	−0,12
17	−0,29	**1,34**	0,28
18	−0,38	**1,81**	−0,52
19	0,21	**1,35**	−0,21
20	0,34	**1,3**	−0,42
21	0,29	**1,3**	−0,32
22	0,15	**1,18**	0,07
23	0,15	**1,61**	0,02
24	0,5	**1,42**	−0,84
25	0,23	**1,93**	−0,95
26	0,52	−0,73	**1,18**

Across the three temperatures tested, a total of 26 proteins were found to change significantly in abundance between the hypoxic and normoxic conditions. All of them were subjected to MALDI TOF/TOF mass spectrometry, but only 11 matched to an EST sequence (Table 2). One EST could not be annotated using BLAST (sequence scallop_rep_c9282) and two other sequences were attributed to hypothetical proteins with no evidence for functions (Contig_RS_4016 and Contig_BAS15570).

**Table 2:** List of proteins identified. List of *Pecten maximus* gill tissue proteins identified by MS/MS, the abundances of which changed significantly between hypoxic and normoxic conditions either at 18 °C or 25 °C (moderate *t*-test paired-comparison, fdr < 0.1, absolute fold change >2).

#Spot	Score	%Cov.	Peptides sequences	Assignation	Acc.	EST
1	21.34	4	HIS^a^FC^b^IS^a^R.C	hypothetical protein CGI_10020036 [*Crassostrea gigas*]	EKC39433	Contig_RS_4016
			SPSSMSWMR.C			
			SFSAPPTPSR.G			
4	75.73	3	ALSSDRHSTVSR.T	Putative ZDHHC-type palmitoyltransferase5 [*Crassostrea gigas*]	EKC28431	Contig_RS_3827
			QPS^a^IT^a^PSR.C			
5	52.72	11	PVLPQS^a^PR.C	NADH-cytochrome b5 reductase-like protein [*Crassostrea gigas*]	EKC35862	Contig_RS_5999
			GSLSRGFS^a^R.G			
11	117.50	7	AAVPSGASTGIYEALELR.G	enolase 3 [*Salpingoeca rosetta*]	XP_004994770	Contig_RS_391
			EANWGVM^c^VSHR.A			
			LGANAILGVSLAVC^b^R.G			
			M^c^GSETYHHKK.G			
12	91.71	2	VIPIFAER.C	Plasma alpha-L-fucosidase [*Crassostrea gigas*]	EKC34412	Contig_RS_12960
			SSRS^a^AS^a^R.T			
13	49.83	2	VLYPLLAR.C	Solute carrier family 15 member 4 [*Crassostrea gigas*]	EKC40000	Contig_RS_4977
			VTMVMGC^b^PRR.C			
			QRC^b^YAS^a^R.T			
14	160.47	4	NSINSGDVYILDLGR.G	gelsolin [*Suberites ficus*]	CAF21863	Contig_RS_292
			NIEVVEVPLSR.A			
			AWDGAGQEPGIQIWR.A			
15	40.72	1	LLYS^a^RY^a^IAR.T	Serine/threonine-protein kinase TBK1 [*Crassostrea gigas*]	EKC21054	Contig_RS_3221
			VWHSC^b^NSR.C			
21	300.00	9	DLYASLQSELK.C	–	–	scallop_rep_c9282
			AIVLFVDGNADDANAAK.G			
22	85.73	2	YQGPFNAEDQGTVR.G	hypothetical protein LOC100635635 [*Amphimedon queenslandica*]	XP_003383336	Contig_BAS_15570
			VYDSVTWVGR.T			
			NACCS^a^SGAPCGAGGAGADLADDCK.A			
23	43.80	3	GNPY^a^IS^a^LISRSQYH.T	protein kinase CK2 alpha catalytic subunit [*Mytilus galloprovincialis*]	CBK38915	Contig_RS_4949
			M^c^PACLS^a^VR.T			

**Notes.**

Modified Amino Acids are indicated as follows:

a Phosphorylation (+79.97)

b Carbamidomethylation of cysteine (+57.02)

c Oxidation of methionine (+15.99).

### Hypoxia response at 18 °C

The arginine content, significantly lower than in normoxia, indicate that animals in hypoxia at 18 °C have probably shifted towards an anaerobic metabolism. Indeed in the anaerobic pathway of *P. maximus* metabolism, arginine can be condensed with pyruvic acid in order to form octopine.

A Casein Kinase 2 alpha catalytic subunit (CK2*α*) was identified as up-regulated under hypoxia at 18 °C (spot 23, Fig. 2, Tables 1 and 2). CK2 is an ubiquitous serine/threonine protein kinase found in eukaryotic cells, involved in a variety of cellular processes including metabolism, signal transduction and transcription (Pinna, 1990). Interestingly, the CK2*α* protein has recently been observed to be accumulated during hypoxia in human culture cells (Mottet et al., 2005). Moreover, some authors proposed a key role of CK2*α* in hypoxia regulation, through the mediation of Hypoxia Inducible Factor-1 activity (HIF-1; Mottet et al., 2005; Hubert et al., 2006). HIF-1 is a major transcriptional regulator of cell responses to reduced oxygen level, and it has been shown to be up-regulated under hypoxia in numerous species (Gorr, Gassmann & Wappner, 2006; De Palma et al., 2007), including bivalves (Kawabe & Yokoyama, 2012). It seems that the activity of CK2*α* increases the transcriptional activity of HIF-1 without increasing HIF-1 at the protein level (Mottet et al., 2005). Indeed, the CK2 protein could also phosphorylate the p53 protein, a competitive inhibitor of HIF-1, thus targeting its degradation through the proteasome (Hubert et al., 2006). The increase of CK2 in hypoxia could thus lead to a down-regulation of p53 and thereby to an enhanced transcriptional activity of HIF-1. Furthermore, CK2 is also known to play a major role in inhibition of apoptosis through phosphorylation of the Bid protein, and subsequent inhibition of the caspases pathway (Yamane & Kinsella, 2005; Ahmad et al., 2008).

Two proteins down-regulated under hypoxia at 18 °C were identified as a NADH cytochrome b5 reductase and a ZDHHC type palmitoyl transferase (spots 5 and 4, respectively, in Tables 1 and 2). NADH-cytochrome b5 reductases are notably involved in the desaturation and elongation of fatty acids, and in cholesterol biosynthesis. These results suggest a decrease in unsaturated fatty acid content along with a decrease in sterol biosynthesis under hypoxic conditions. Of note, such decrease was observed in metabolomic studies in yeast (Gleason et al., 2011), rat (Bruder, Lee & Raff, 2004) and human aorta (Filipovic & Rutemöller, 1976). Fatty acid metabolism under hypoxia is poorly known. Nevertheless, such a shift in fatty acid composition could have deep implications for membrane structure and/or energy metabolism. ZDHHC-type palmitoyl transferases catalyse the transfer of palmitate, a 16-carbon saturated fatty acid, on proteins. Their roles in hypoxia response are difficult to assess, as palmitate was reported to be linked to more than 100 proteins (Resh, 2006). Palmitoylation is a reversible modification of proteins mainly associated with their anchoring in biological membranes. In particular, this post-translational modification plays an important role in regulating ion channel localization and activity (El-Husseini & Bredt, 2002). Indeed, localization and activity is modulated by palmitoylation directly on the ion channels, many of which are also receptors, as well as on the scaffolding proteins that bind to the channels (Smotrys & Linder, 2004). Therefore, the down-regulation of palmitoyl transferase could reflect a change in a signalling pathway under hypoxic conditions.

### Hypoxia responses at 25 °C

The proteomic pattern of scallop gills at 25 °C under hypoxia differed strikingly from that observed at 18 °C. Only 11 spots were significantly changed at 25 °C, and only one protein is shared with the 18 °C hypoxia response. It is noteworthy that at 25 °C, *Pecten maximus* have been reported to be out of its optimal thermal window (Artigaud et al., 2014c) and the observed differences may be explained by the heat stress experienced at this temperature. Indeed, it could be hypothesized that the severe heat stress may prevent animals from developing the whole hypoxia response, which is consistent with results obtained at the enzymatic level (Fig. 1) and with the mortalities observed during this experiment. Given the level of arginine contents (Fig. 1A) and ODH activity (Fig. 1B), animals maintained at 25 °C in normoxic condition may already have experienced a limitation of their aerobic capacities through cellular hypoxia only caused by heat stress. Therefore, their capacities to cope with new stressful situation, such as hypoxia, may be reduced. As 25 °C is outside of the optimal thermal window for scallop, the animals presumably already encountered a maximal oxygen demand, so as the animals could probably not further modify their metabolism to acclimate to hypoxia. Similarly, studies on the oyster *Crassosstrea gigas* also found an impact on metabolism when exposed to multiple stressors (Lannig et al., 2010; Timmins-Schiffman et al., 2014). In a recent study, proteomic profiles of oysters’ gills exposed to a mechanical stress were modified if they were previously exposed to a chronic low pH (Timmins-Schiffman et al., 2014).

Among the proteins differentially expressed during hypoxia at 25 °C, only 1 protein was up-regulated, but it could not be identified by mass spectrometry. 5 of the 10 down-regulated proteins under hypoxia at 25 °C were formally identified by mass spectrometry (spots 11–15, Tables 1 and 2).

Two of the down-regulated identified proteins could be involved in signalling, i.e., the protein TANK binding kinase 1 (TBK1; spot 15, Tables 1 and 2) and a channel cotransporter of oligopeptides (Solute carrier family 15 member 4; spot 13, Tables 1 and 2). TBK1 promotes the TNF-induced NF-*κ*B activation by phosphorylating I-kB, thus promoting its degradation and the subsequent activation of the NF-*κ*B transcriptional regulator (Tojima et al., 2000). In addition, TBK1 was suggested to act as an inhibitor of apoptosis, as RNA interference analyses showed an increase in apoptosis induced by TNF (Fujita et al., 2003). In our experiment, TBK1 were found to be down-regulated, which could cause an increase in apoptosis of gill cells at 25 °C under hypoxic conditions.

Another down-regulated protein observed at 25 °C under hypoxia, gelsolin (spot 14, Tables 1 and 2), is an actin binding protein involved in the regulation of actin dynamics (Li et al., 2012). Increasing evidence showed that gelsolin is a multifunctional regulator of cell metabolism involved in multiple mechanisms, independently of its actin regulatory functions (Sun et al., 1999; Silacci et al., 2004). Among these functions is the pro-apoptotic activity of gelsolin through the gelsolin-HIF1*α*-DNase I pathway (Li et al., 2009). A decrease in gelsolin could thus be anti-apoptotic, contrasting with the decrease of TBK1, which could be pro-apoptotic. Apoptosis is a complex phenomenon and a matter of balance between “life” and “death” signals (Spyridopoulos et al., 1997). As an example, the TNF activation of the NF-*κ*B pathway was shown to promote either pro- or anti-apoptotic effects, depending on the nature of the stimulus (Kaltschmidt et al., 2000). Therefore, further studies will be needed in order to elucidate the regulation of apoptosis under hypoxic conditions at 25 °C in *P. maximus* gills.

Two other down-regulated proteins identified are involved in energy metabolism: enolase (spot 11, Tables 1 and 2) and alpha-L-fucosidase (spot 12, Tables 1 and 2). Enolase is an enzyme involved in glycolysis, catalysing the conversion of 2-phosphoglycerate (2-PG) to phosphoenolpyruvate (PEP). Specifically enolase 3 could be linked to glycogen utilization, as a mutation of the gene encoding enolase 3 (ENO3) in humans has been reported to trigger glycogen storage disorder (Comi et al., 2001). Down-regulation of enolase 3 in hypoxic conditions has been observed at the protein level in trout (Wulff et al., 2012) and rats (De Palma et al., 2007). Down-regulation of enolase 3 might reflects an attempt to limit energy consumption by shifting in a hypometabolic state. Anaerobic metabolism is the major source of energy for marine organisms under hypoxia (Grieshaber et al., 1994). Therefore, as the transition to fermentative metabolism generally implies increased glycolytic fluxes to produce high amounts of pyruvate, the down-regulation of glycolytic enzymes should not be expected in hypoxia.

The last down-regulated protein under hypoxia at 25 °C which has been identified is alpha-L-fucosidase (spot 12, Tables 1 and 2). Fucosidases are enzymes associated with carbohydrate metabolism, as they remove terminal L-fucose residues present on the oligosaccharide chains of glycoconjugates (Johnson & Alhadeff, 1991). A wide variety of conjugates can be fucosylated, and fucosidases act on glycoproteins, glycolipids and glycans (Johnson & Alhadeff, 1991; Becker & Lowe, 2003). Fucosidases are located in lysosomes where their actions are required as the first step in degradation of glycoproteins containing complex N-linked chains (Freeze, 1999). Therefore, a decrease in hypoxia at 25 °C of alpha-L-fucosidases could be part of an energy saving strategy by reducing the protein turnover.

Overall, data obtained from animals under hypoxic conditions at 25 °C suggest a general severe crisis, implying attempts of energy savings, rather than a specific response to hypoxia. Apoptosis phenomena might also be involved, but further studies are needed to elucidate the pro-and anti- apoptotic signals in these conditions.

## Concluding Remarks

This study highlights a strong temperature effect on the response of *Pecten maximus* to hypoxia. Different proteomic signatures between normoxic and hypoxic conditions were observed at 18 °C and 25 °C. No changes in the proteomic phenotype between normoxia and hypoxia of gills tissue was observed at 10 °C suggesting that the low energy demand due to hypoxia at this temperature did not require extra proteins adjustments. Additionally, our enzymatic assays indicated that *P. maximus* did not switch to anaerobic metabolism under a 24-hours hypoxia at 10 °C. At 25 °C, the enzymatic assays did not show any difference between normoxia and hypoxia, suggesting that the bivalves were already under anaerobic metabolism in normoxic condition. The only temperature treatment used in this study allowing to observe a hypoxia proteomic signature is the one performed at 18 °C, as data obtained at 25 °C suggest attempts to save energy more than a specific response to hypoxia. In all, 26 protein spots were significantly changed at either 18 °C or 25 °C. We could sequence peptides from 11 proteins, and assign functions for 8 of them. The results suggested a down regulation of some parts of the energetic metabolism, and a role for apoptosis in the hypoxia response following thermal acclimation. Several proteins could be linked to HIF related metabolisms. Further studies should determine the exact role of these proteins as effectors of the response to hypoxia, with a special focus on their possible interaction with HIF and with apoptosis.

## Supplemental Information

10.7717/peerj.871/supp-1 File S1 Supplemental file 1

10.7717/peerj.871/supp-2 File S2 Raw volume of 2-DE gels This a .csv file with all the raw volume from 2-DE gels issued from the image software.

## Additional Information and Declarations

### Data Deposition

The following information was supplied regarding the deposition of related data: The 2-DE Gels from the experiment are available in FigShare: http://figshare.com/articles/2_DE_Gels_of_Pecten_maximus_gills_at_three_different_temperatures_and_two_O2_levels_normoxia_or_hypoxia_/1328460. The database we used for mass spectrometry is also available in FigShare: http://figshare.com/articles/Pecten_maximus_EST_database/1328466.
